# Influence of Artefact Correction and Recording Device Type on the Practical Application of a Non-Linear Heart Rate Variability Biomarker for Aerobic Threshold Determination

**DOI:** 10.3390/s21030821

**Published:** 2021-01-26

**Authors:** Bruce Rogers, David Giles, Nick Draper, Laurent Mourot, Thomas Gronwald

**Affiliations:** 1 College of Medicine, University of Central Florida, 6850 Lake Nona Boulevard, Orlando, FL 32827-7408, USA; 2 Lattice Training Ltd., Chesterfield S41 9AT, UK; dave@latticetraining.com; 3 School of Health Sciences, College of Education, Health and Human Development, University of Canterbury, Christchurch 8041, New Zealand; nick.draper@canterbury.ac.nz; 4 EA3920 Prognostic Factors and Regulatory Factors of Cardiac and Vascular Pathologies, Exercise Performance Health Innovation (EPHI) Platform, University of Bourgogne Franche-Comté, 25000 Besançon, France; laurent.mourot@univ-fcomte.fr; 5 Division for Physical Education, National Research Tomsk Polytechnic University, Lenin Ave, 30, 634050 Tomsk Oblast, Russia; 6 Department of Performance, Neuroscience, Therapy and Health, MSH Medical School Hamburg, Faculty of Health Sciences, University of Applied Sciences and Medical University, Am Kaiserkai 1, 20457 Hamburg, Germany; thomas.gronwald@medicalschool-hamburg.de

**Keywords:** heart rate variability, detrended fluctuation analysis, ventilatory threshold, aerobic threshold, intensity distribution, artefact, endurance exercise, heart rate monitors, wearables

## Abstract

Recent study points to the value of a non-linear heart rate variability (HRV) biomarker using detrended fluctuation analysis (DFA a1) for aerobic threshold determination (HRVT). Significance of recording artefact, correction methods and device bias on DFA a1 during exercise and HRVT is unclear. Gas exchange and HRV data were obtained from 17 participants during an incremental treadmill run using both ECG and Polar H7 as recording devices. First, artefacts were randomly placed in the ECG time series to equal 1, 3 and 6% missed beats with correction by Kubios software’s automatic and medium threshold method. Based on linear regression, Bland Altman analysis and Wilcoxon paired testing, there was bias present with increasing artefact quantity. Regardless of artefact correction method, 1 to 3% missed beat artefact introduced small but discernible bias in raw DFA a1 measurements. At 6% artefact using medium correction, proportional bias was found (maximum 19%). Despite this bias, the mean HRVT determination was within 1 bpm across all artefact levels and correction modalities. Second, the HRVT ascertained from synchronous ECG vs. Polar H7 recordings did show an average bias of minus 4 bpm. Polar H7 results suggest that device related bias is possible but in the reverse direction as artefact related bias.

## 1. Introduction

Several heart rate variability (HRV) indexes have shown potential as indicators of exercise effort and training intensity zone demarcation [1,2]. Recently, a non-linear HRV index of fractal correlation properties, the short-term scaling exponent alpha 1 of detrended fluctuation analysis (DFA a1) has received particular attention as both an indicator of autonomic nervous system regulation as well as an overall marker of organismic demand [3]. This measurement is based on fractal dynamics and self-similarity of the cardiac beat to beat pattern [4]. DFA a1 has been shown to decline as work rates rise, starting from strongly correlated patterns at levels below the first ventilatory threshold, transitioning through values representing uncorrelated, less complex behavior at moderate to high work rates, then finally showing anticorrelated and random patterns at the highest intensities [3,5,6]. 

Recent work indicates that when exercise intensity reaches the first ventilatory threshold during an incremental treadmill ramp protocol, DFA a1 values reach 0.75, corresponding to the midway between well correlated fractal beat patterns (between order and disorder) and those of uncorrelated behavior [7]. Since this study utilized interbeat data recorded by a research grade ECG with little to no artefact, it is unclear if these results would be applicable with higher artefact presence as well as using a typical chest belt recording device. In view of the prospect of using a heart rate variability threshold (HRVT) derived from DFA a1 behavior during an incremental exercise ramp for training intensity distribution, an expansion of this concept with consumer wearable devices would be helpful. 

The two most obvious issues that could alter the validity and reproducibility of the HRVT from the laboratory to common practice are the possibility of bias induced by artefact presence with correction and that of an alternate recording device. If the DFA a1 index is to be a robust marker for intensity zone demarcation, consistency of measurement standards is essential for both comparing the basic research done as well as for athletes and patients using this for exercise and training monitoring. The typical method of recording the cardiac interbeat interval in many settings will be via a chest belt device such as the Polar H series (Polar Electro Oy, Kempele, Finland). Chest belt recording systems have been shown to accurately measure heart rate during dynamic exercise [8] but the accuracy of HRV indexes while using these devices can be variable [9,10,11,12,13,14,15]. Therefore, to support the usage of a DFA a1 based HRVT for general application, the effects of artefact as well as a comparison to a consumer chest belt device is necessary.

Artefacts are commonly present in many RR recording sessions and may be more numerous during dynamic exercise at higher intensities when chest movements are involved, i.e., typically running [11]. This may be due to intrinsic heart rate irregularities, false beats related to spurious noise and missed beats due to erratic chest belt contact. Missed beat artefacts have been shown to be the most common variety seen during dynamic exercise [11]. In order for HRV analysis software to calculate the various time domain, frequency domain and non-linear indexes, artefact correction algorithms have been devised [16]. If artefact correction is not applied, even a single artefact can significantly alter HRV parameters for a short measurement window [17,18]. Study results are mixed with respect to the effects that correction methods have on HRV index accuracy when compared to a parallel ECG recording without artefact [11,19,20,21]. Artefact correction algorithms generally encompass two separate actions. The initial step is identification of what comprises a missed or aberrant beat, based on an approximation of the running pattern of the interbeat interval. In the case of a missed beat, the next action is placing an artificial beat into the gap in the RR series depending on a predetermined formula. This formula is based on various interpolation models (e.g., linear, cubic spline) of the past and future RR series [16]. Potential deviation from the true position of the missed beat can be due to inappropriate designation of aberrancy and/or failure to correctly model the interpolation.

A popular HRV software package, Kubios HRV Software (Version 3.4.1, Biosignal Analysis and Medical Imaging Group, Kuopio, Finland) has two methods of artefact correction. The freely available version of Kubios employs a cubic spline interpolation method [22] that is termed “Threshold” correction. The “Threshold” level (Very Low, Low, Medium, Strong, Very Strong or Custom) is based on the time deviation from an anticipated beat pattern derived from a running average of the prior RR intervals. Depending on the time difference from this average, the proposed aberrant beats are corrected by a cubic spline formula. The “Premium” version of Kubios can employ this method but is also capable of a newer correction technique called “Automatic” [23]. The “Automatic” method uses a different modeling approach to determine beat aberrancy (without the ability to adjust beat to beat time deviation tolerance). In addition, it appears to use linear interpolation to replace the missing interval, inserting a replacement beat at the halfway time point between the valid RR intervals. Thus, two different correction methods are potentially available. Since the newer, “Automatic” method has only recently become available, many previous investigations into artefact correction have not evaluated what, if any consequence is introduced by this process. Giles and Draper [11] have shown significant bias in both non-linear and time domain HRV indexes with the “threshold” artefact correction method of Kubios (Version 2.1). Other studies using various models of either artificially inserted artefact [19,20] and animal models [21] have shown variable effects on non-linear indexes. Some investigations have concluded that artefact correction methods yielded similar index values but did not simultaneously record an artefact free comparator and have just compared artefact correction methods to themselves [24]. Given the interest of DFA a1 as an exercise intensity marker, it would be essential to know whether artefact impacts the DFA a1 index and at what rates do the measurements lose validity. Perhaps more importantly, what are the physiologic correlates of artefact presence and correction methodology during dynamic exercise? Since this index declines with increasing exercise intensity [3,25,26,27,28,29], does the presence of artefact and subsequent correction alter the DFA a1 vs. intensity relationship? 

Consequently, the purpose of this study was threefold:

1. Investigate the degree of bias in the DFA a1 index caused by the presence of missed beat artefact by the automatic and threshold correction modalities of Kubios (Version 3.4.1). Since research groups and consumers will use this popular HRV software program but only the threshold method is available in the free version, both artefact correction methods will be examined.
2. To explore the importance of missed beat artefact on the location of the HRVT. In other words, as a physiologic marker related to the aerobic threshold [6,7], does the calculated HRVT heart rate shift by the presence of artefact?
3. Compare DFA a1 data gathered from a research grade ECG to that obtained from a Polar H7 recording device. Although a direct comparison of artefact free tracings from both a chest belt and ECG are easily accomplished for subjects at rest, it is generally impractical to expect artefact free chest belt recording during high intensity exercise [11]. In lieu of this limitation, we will not attempt to systematically compare artefact free segments of Polar H7 vs. ECG data. Instead, a realistic use case comparison will be done such as evaluation of the HRVT heart rate between the devices when worn simultaneously.

## 2. Methods

### 2.1. Participants

Seventeen male volunteers aged 19 to 52, without previous medical history, current medications or physical issues were tested. Participants were informed of the potential testing risks and institutionally approved consent was given. Approval for the study was granted by the University of Derby, UK [LSREC_1415_02] and conformed to the principles of the Declaration of Helsinki. Participants did not consume caffeine, alcohol or any stimulant for the 24 h before testing, nor was there any current tobacco usage. Background data for each participant included, age (29 ± 10 y), body weight (77 ± 9 Kg) and training volume in hours per week has been described [7]. All testing was done in the afternoon and at least 3 h post meal. No exercise was performed the day prior to the test. Two participants with excessive cardiac ectopy (frequent atrial premature beats and atrial trigeminy) during testing were excluded from HRVT analysis.

### 2.2. Exercise Protocol

Participants performed an incremental maximal cardiopulmonary exercise test on a motorized treadmill (Woodway, Birmingham, UK). The treadmill was set for the Bruce protocol with increases in speed and inclination from 2.7 km/h at ten percent grade, increasing by 1.3 km/h and two percent grade every 3 min until volitional exhaustion. A fan was used for cooling.

### 2.3. Gas Exchange Testing and Calculation of the First Ventilatory Threshold

Gas exchange kinetics were recorded continuously using a breath-to-breath metabolic cart (Metalyzer 3B; Cortex Biophysik GmbH, Leipzig, Germany). In addition, a Polar H7 (Polar Electro Oy, Kempele, Finland) was wirelessly paired to the Metalyzer cart for the purpose of beat to beat HR recording concurrent with gas exchange data. Analysis of the above parameters were done to derive the first ventilator threshold (VT1), maximal oxygen uptake (VO_2MAX_) and VO_2_ vs. time as previously reported and documented [7]. VT1 was determined by the excess CO_2_ method [30]. Two experienced observers confirmed VT1 results independently.

### 2.4. RR Measurements and Calculation of DFA a1 Derived Threshold

A 3-lead ECG (MP36; Biopac Systems Ltd., Essen, Germany) with a sampling rate of 1000 Hz was used to record the participant’s ECG/RR times series. Electrodes were placed in the CM5 distribution after appropriate skin cleansing and shaving if necessary. Sample data from the MP36 was saved as acq files. In addition, simultaneous recording with the Polar H7 (Polar Electro Oy, Kempele, Finland) chest belt was done. ECG and Polar H7 data files for each participant were imported into Kubios HRV Software (Version 3.4.1, Biosignal Analysis and Medical Imaging Group, Department of Physics, University of Kuopio, Kuopio, Finland). Kubios preprocessing settings were at the default values including the RR detrending method which was kept at “Smoothn priors” (Lambda = 500 [22]). For DFA a1 estimation, the root mean square fluctuation of the integrated and detrended data is measured in observation windows of different sizes. The data are then plotted against the size of the window on a log-log scale. The scaling exponent represents the slope of the line, which relates (log) fluctuation to (log) window size [6]. DFA a1 window width was set to 4 ≤ *n* ≤ 16 beats. 

### 2.5. Artefact Addition to ECG and Influence on DFA a1

Each participant’s incremental ramp ECG was examined with Kubios software including visual inspection to determine sample quality, noise, arrythmia and missing beat artefact. Non-overlapping, consecutive, non-artefact containing 2-min measurement windows were identified for each participant over their entire ramp. The chosen method of artefact introduction is based on that used by Stapelberg et al. [20], which relies on manually removing random beats from the already recorded artefact free series to achieve a desired “missed beat” artefact percentage. A major advantage of this method is the elimination of timing synchronization errors between paired comparisons as the same data windows are evaluated. Since we are also interested in possible recording device differences, a cut beat approach would eliminate any potential across device bias as well. Using the Kubios RR editing tool (Premium Version), random RR peaks were removed from the measurement windows to result in 1, 3 and 6% total missed beats per window. The HRV data were saved as text files for baseline (no artefact, NA) each artefact manipulation step and correction method applied, 1% induced artefact with automatic correction (1% AC), 1% induced artefact with medium threshold correction (1% MC), 3% induced artefact with automatic correction (3% AC), 3% induced artefact with medium threshold correction (3% MC), 6% induced artefact with automatic correction (6% AC) and 6% induced artefact with medium threshold correction (6% MC). The “medium” setting for the threshold correction method was chosen based on it being the Kubios default choice as well as evidence of it being optimal for adult populations [31].

### 2.6. Influence of Artefact on ECG Derived HRVT

A time varying data file was generated for each participant based on a 2-min measuring window with a recalculation (grid interval) every 5 s throughout the test. As discussed previously [7], a DFA a1 value of 0.75 was chosen for the detection of a HRV derived threshold (HRVT) based on this being the midpoint between a fractal behavior of the HR time series of 1.0 (seen with very light exercise) and an uncorrelated value of 0.5 which represents uncorrelated/random behavior (seen with high intensity exercise). The following procedure was used to indicate at what level of running intensity (represented by heart rate) the DFA a1 would cross a value of 0.75: First, ECG data from each 2-min rolling window was used to plot the average HR vs. DFA a1. The HR at which DFA a1 equaled 0.75 was found using a linear regression through the rapid change section of DFA a1 values of about 1.0 to 0.5 or below, with a subsequent equation for HR and DFA a1. Using a fixed variable of DFA a1 equals 0.75, the resulting HR was obtained from the equation. Only 10 participants with zero artefact in the HRVT measurement portion qualified for evaluation in the artefact intervention comparison. This was done by repeating the HRVT process for each artefact level and correction modality. The beginning and end time of the linear regression was exactly matched for each participant over each artefact intervention.

### 2.7. Influence of Polar H7 on HRVT

**Figure 1:**
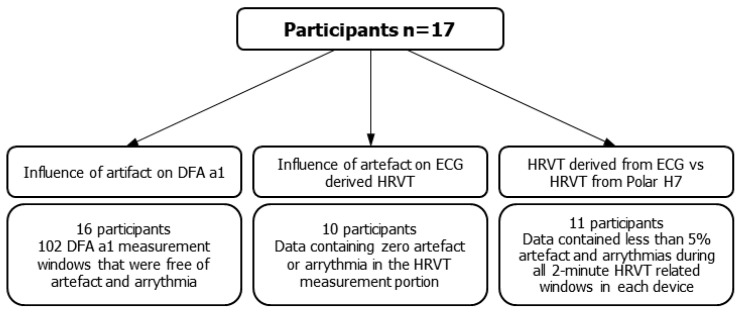
Inclusion criteria, numbers of included participants and data intervals for the threefold purpose of this study.

All participants had Polar H7 recorded RR series analysis by Kubios software. HRVT and artefact percentage was determined using the above-mentioned technique. To achieve a sufficient sample size for meaningful comparison, the following limits to artefact were set for study inclusion. Both the Polar and ECG derived data contained less than 5% artefact during all 2-min HRVT related windows, which yielded 11 participants who met these criteria. A summary of each inclusion criteria is shown in Figure 1.

### 2.8. Statistics

Statistical analysis was performed for the tested variables using standard methods for the calculation of means, medians and standard deviations (SD). Normal distribution of data was checked by Shapiro-Wilk testing and visual inspection of data histograms. The correlation between the non-overlapping 2-min segments (artefact containing data against the DFA a1 NA), HRVT NA vs. HRVT artefact intervention and HRVT Polar H7 vs. HRVT ECG was assessed using Pearson’s r correlation coefficient, standard error of estimate (SEE), coefficient of determination (R^2^) and Bland Altman plots with limits of agreement [32]. The size of Pearson’s r correlations evaluated as follows; 0.3 ≤ r < 0.5 low; 0.6 ≤ r < 0.8 moderate and r ≥ 0.8 high [33]. Estimate of the median difference between NA and artefact segments of the non-overlapping 2-min windows was calculated using the Hodges Lehmann shift method along with Wilcoxon testing of paired groups in view of potential outlier values [34,35]. Although agreement between groups was assessed by Bland Altman analysis, if proportional bias was detected, regression-based calculation of mean differences and limits of agreement were performed [36,37]. Bland Altman mean differences for 2-min segment comparisons was expressed as percentage bias (difference/mean × 100). Conventional Bland Altman analysis was performed to determine agreement between HRVT groups. Paired t testing was used for comparison of HRVT for both artefact and device conditions. For all tests, the statistical significance was accepted as *p* ≤ 0.05. Cohen’s d was used to denote effect sizes (small effect = 0.2, medium effect = 0.5, large effect = 0.8; [38]). Analysis was performed using Microsoft Excel 365 with Real Statistics Resource Pack software (Release 6.8) and Analyse-it software (Version 5.66).

## 3. Results

### 3.1. Gas Exchange

Detailed gas exchange results and heart rate at HRVT has been previously reported (7). Mean participant VO_2MAX_ was 56 mL/kg/min (±10), VT1 was reached at 70% (±6) of VO_2MAX_, heart rate at VT1 by gas exchange was 152 bpm (±21), HRVT heart rate 154 bpm (±20) and artefact 0.6% (±0.9) during the HRVT relevant RR series.

### 3.2. Artefact Addition to ECG Recording and Influence on DFA a1with 1, 3 and 6% Artefact

**Figure 2:**
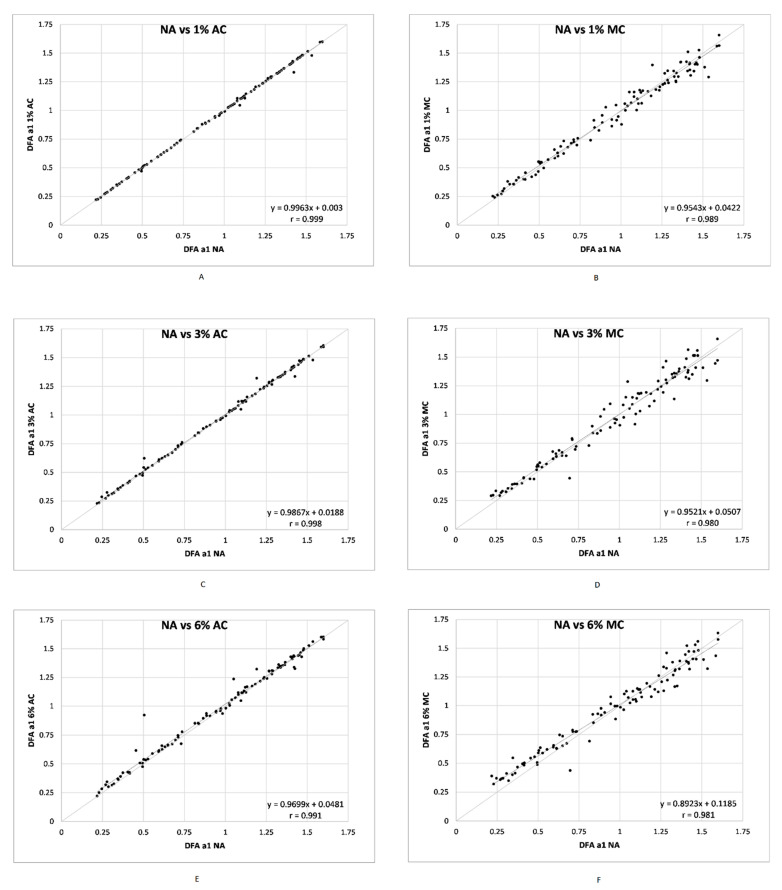
Regression plots for all ECG derived DFA a1 NA vs. DFA a1 for each artefact condition and correction method. (**A**) vs. DFA a1 1% AC; (**B**) vs. DFA a1 1% MC; (**C**) vs. DFA a1 3% AC; (**D**) vs. DFA a1 3% MC; (**E**) vs. DFA a1 6% AC; (**F**) vs. DFA a1 6% MC. Bisection lines in light gray. Slope and Pearson’s r shown in bottom right of each plot.

**Figure 3:**
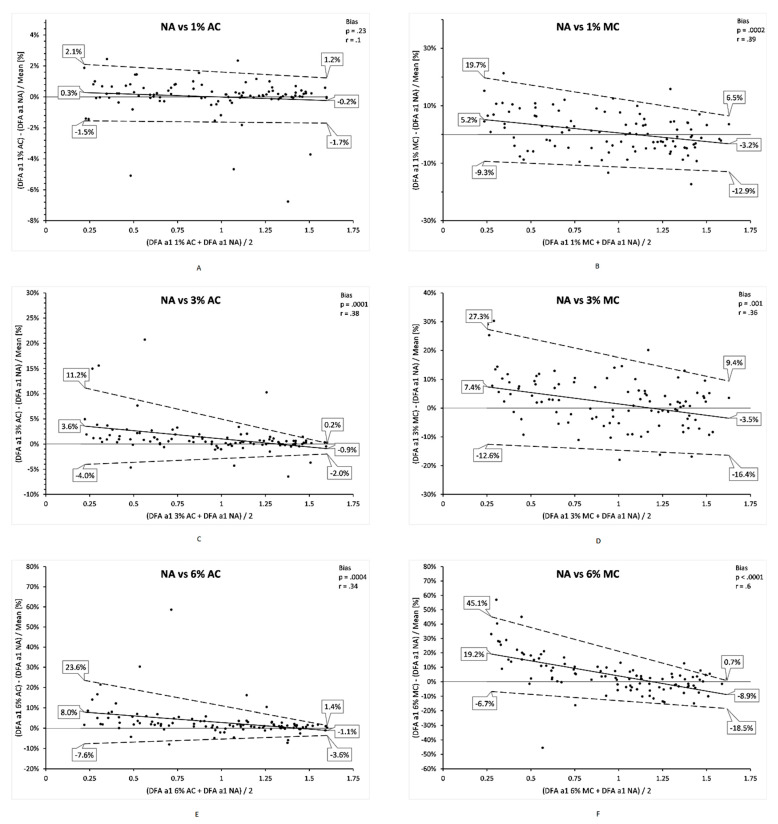
Bland Altman analysis of ECG derived DFA a1 NA vs. DFA a1 for each artefact condition and correction method using regression based mean and standard deviations. (**A**) vs. DFA a1 1% AC; (**B**) vs. DFA a1 1% MC; (**C**) vs. DFA a1 3% AC; (**D**) vs. DFA a1 3% MC; (**E**) vs. DFA a1 6% AC; (**F**) vs. DFA a1 6% MC. Center solid line in each plot represents the mean bias (difference) between each paired value as relative percent (difference/mean × 100). The top and bottom dashed lines are 1.96 standard deviations from the mean difference. Pearson’s r for the regression line of bias with *p* value shown on top right of each plot.

There were 102 DFA a1 measurement windows from 16 participants that were free of artefact. Table 1 contains the mean, standard deviation, median, minimum, maximum, along with paired artefact group comparisons to DFA a1 NA including adjusted Hodges Lehmann median shift, Wilcoxon paired testing and Pearson’s r. Regression plots for DFA a1 NA vs. each artefact condition and correction method are shown in Figure 2. Correlation values ranged from 0.999 to 0.980, with AC generally higher than MC for each artefact group. Bland Altman difference charts were done for each condition (1, 3, 6% artefact AC and MC) as seen in Figure 3. Analysis of proportional bias (change in the bias over the DFA a1 range), heteroscedasticity (change in scatter of differences) was evaluated with regression of either mean differences and/or limits of agreement respectively according to the recommendations of Ludbrook [36]. 

### 3.3. Influence of Artefact Condition and Correction Method on the ECG Derived HRVT

**Figure 4:**
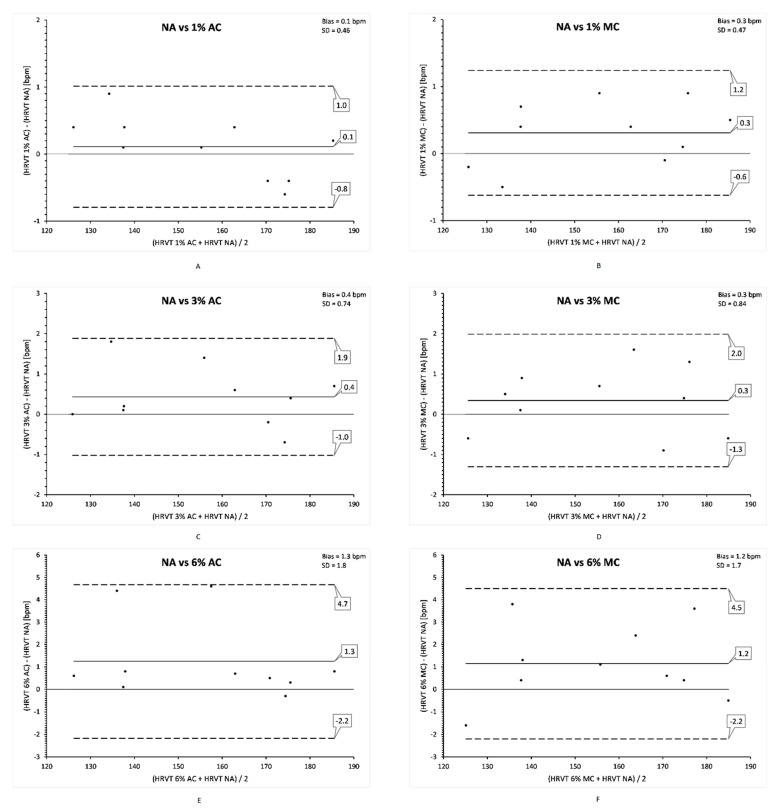
Bland Altman analysis of ECG derived HRVT NA vs. HRVT for each artefact condition and correction method. (**A**) vs. 1% AC; (**B**) vs. 1% MC; (**C**) vs. 3% AC; (**D**) vs. 3% MC; (**E**) vs. 6% AC; (**F**) vs. 6% MC. Center solid line in each plot represents the mean bias (difference) between each paired value. The top and bottom lines are 1.96 standard deviations from the mean difference. Net bias with standard deviation (SD) in top right portion of each plot with standard deviation (SD) in top right portion of each plot.

The impact of artefact condition and correction method on the calculated, ECG derived HRVT for each participant is shown in Table 2. Although small differences in mean HRVT were seen between artefact groups and NA, only the 6% AC group reached significance on t testing. Bland Altman difference analysis is shown in Figure 4. Artefact levels of 1, 3 and 6% caused an average bias of 0.1, 0.4 and 1.3 bpm using AC and 0.3, 0.3 and 1.2 bpm using MC method. Limits of agreement for the three groups were −1 to 0.8, 1 to −1.9, 2.2 to −4.7 bpm for AC and 0.6 to 1.2, 1.3 to 2.0 and 2.2 to −4.5 for MC respectively. All points were within the 2 standard deviation limits of agreement.

### 3.4. HRVT Derived from ECG vs. HRVT Derived from Polar H7

**Figure 5:**
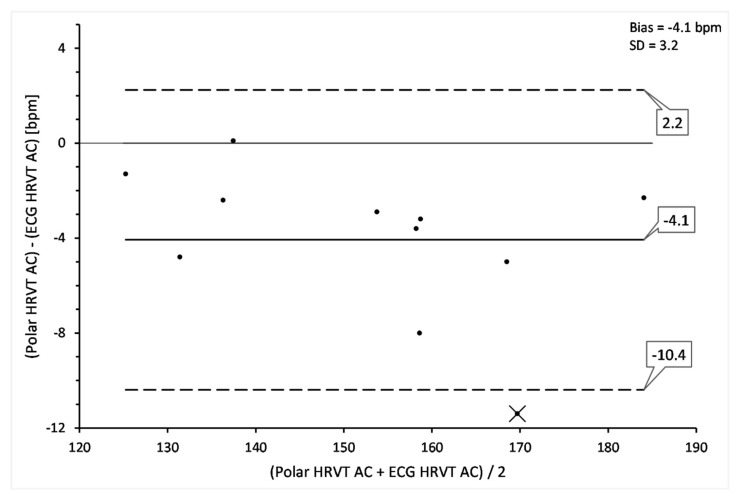
Comparison of heart rate at HRVT between ECG vs. Polar H7 data using Bland Altman analysis. The one outlier (labeled as X) represents the only participant with no artefact in both ECG and Polar H7 time series. Center line represents the mean bias (difference) between each paired value. The top and bottom lines are 1.96 standard deviation from the mean difference. Bias and standard deviation (SD) listed in upper right corner.

**Figure 6:**
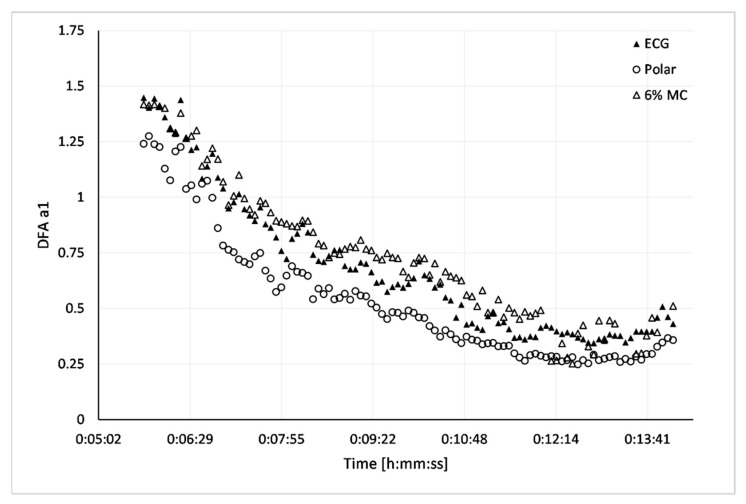
Time-varying analysis (window width: 120 s, grid interval: 5 s), DFA a1 for matched time series containing no artefact in one representative participant, ECG (solid triangle), Polar H7 (open circle), ECG 6% MC (open triangle).

The HRVT derived from the Biopac 36 ECG was compared to that of the HRVT obtained from Polar H7 data. Both times series contained less than 5% artefact during each 2-min measurement window. The AC method was used for all RR time series based on the superior bias seen in comparison to MC. Results are presented in Table 3. Bland Altman difference analysis is shown in Figure 5. There was a 4 bpm difference in the mean heart rate between the ECG derived HRVT and the Polar H7 data series (*p* = 0.002, d = 0.228). The bias between recording devices is best illustrated in one participant who had zero artefacts in both the ECG and Polar sequence shown (Figure 6). In this individual’s RR recordings, Pearson’s r correlation between time matched pairs of heart rate for the ECG vs. Polar H7 device heart rate was 0.999 with a slope of 1.0 confirming a near perfect synchronization of the HRV sequence. Time varying display of DFA a1 showed a lower Polar H7 value at almost every measurement window. It should also be noted that this subject had the largest HRVT difference between devices. As will be discussed later, the bias seen here is in the opposing direction from artefact correction as seen with the ECG based 6% MC. 

## 4. Discussion

The purpose of this report was to explore three questions, does bias occurs in the non-linear HRV index DFA a1 from missed beat artefact correction, is there an impact of these artefacts (with correction) on the aerobic threshold related HRVT and does an alternate recording device produce comparable HRVT results to a research grade ECG. The importance of evaluating a physiologic biomarker, the HRVT revolves around the clinical consequence of any noted bias. Even though some numerical bias may occur, it would be important to ascribe a functional relevance to this by leveraging the relationship of DFA a1 decline with increasing exercise intensity [3]. Prior HRV studies have generally not attempted to associate the effect of artefact correction on the corresponding physiological response of interest. It is likely that typical users interested in exercise monitoring will be using a chest belt device with potential missed beat artefact in the recording. Therefore, in view of our recent study linking a DFA a1 derived HRVT with the first ventilatory threshold [7] an examination of both artefact and recording device bias is warranted before extrapolating these results to real world consumer usage. 

Our results show that the artificial addition of between 1 and 6% missed beat artefact did induce progressive amounts of bias into the calculation of DFA a1. Using either the AC or MC methods from Kubios Software at the 1 or even 3% level produced similar results in the median DFA a1 index with small amounts of bias in the Bland Altman analysis. Even at the 6% level, AC methodology produced reasonable results, albeit with some proportional bias and more outliers. However, at 6% MC, both a large number of outliers and substantial proportional bias was seen. It should be noted that previous reports showing high levels of bias in non-linear HRV during exercise used the medium threshold correction method [11]. As the level of artefact rose, AC methodology demonstrated both less bias as well as a reduction in scatter between the differences. Review of the regression analysis for each artefact level and correction method also confirms the trivial effects of 1% artefact especially with AC usage. However, at higher artefact levels, artefact correction falsely raised the DFA a1 index, particularly across its low, uncorrelated and anticorrelated range (interbeat random behavior). Calculation of the HRVT was minimally affected by either 1 or 3% artefact using either correction method. At the 6% artefact level, both AC and MC had similar results, although with small degrees of bias reaching just over 1 bpm. It is interesting to speculate why large amounts of the proportional bias seen with the 6% MC group, had marginal effects on the HRVT. The HRVT is calculated for DFA a1 values between 1 and 0.5, however even with 6% artefact, values just under 1 are minimally affected, values near 0.5 moderately affected, with most of the bias below this point. After reviewing the Bland Altman analysis for the 2-min window comparisons, one could conclude that the addition of 3% missed beat artefact would have minimal, if any effect on the HRVT unless the index was skewed by a random outlier value. At the 6% artefact level, one would also expect reasonable concordance with artefact free data. However, there was a higher occurrence of outliers, especially using MC which could indicate the need to repeat the HRVT assessment for confirmation.

A surprising finding of this study was the discordance of DFA a1 measured by the research grade ECG and that of the Polar H7 chest belt. Although the absolute bias was about minus 4 bpm for the Polar H7 chest belt, this may not be of major clinical relevance especially given the low effect size, however the limits of agreement were about ±6 bpm, corresponding to a net negative 10 bpm. Other studies looking at HRV validation across devices similar to the Polar H7 have not reported similar findings [9,10,12,13,14,15]. Several reasons for this discrepancy are possible. Most device comparisons are done at rest and not during dynamic exercise as was done here. One study using similar device comparisons [16] did not perform detailed analysis of non-linear HRV over the spectrum of exercise intensities. An interesting scenario that may help explain the lack of cross device discrepancy reported in the literature may relate to the bias negation caused by artefact correction effects. For example, at higher artefact levels, especially when corrected by MC, the proportional bias leads to a higher than expected DFA a1 especially at the lower, anticorrelated ranges below 0.5. Therefore, the downward bias in the Polar H7 recording (especially in the uncorrelated range) would be somewhat offset by the effects of expected artefact at higher intensity. This is best understood with the simultaneous measurement of both heart rate and HRV in one specific case (Figure 6). This participant, who had no artefact in both device recordings for a substantial length of time, clearly shows that DFA a1 calculated from the Polar H7 RR time series was consistently lower than that of ECG based results, especially at uncorrelated ranges. The parallel ECG recording with 6% MC data series was included to show how a potential neutralization of bias could occur with coexistent artefact. The negative bias of the Polar H7 would be counterbalanced by the positive bias from artefact correction leading to DFA a1 values near the correct range. Proper synchronization of this time series was corroborated by a plot of the heart rate recorded by each device showing near perfect agreement and correlation. 

Our results did show differences between correction methods (AC vs. MC) for DFA a1 during the 2-min measurement window comparisons. Both methods use a somewhat similar missing beat interpolation approach, namely cubic spline with “Threshold” or linear with “Automatic” correction. They do use different strategies for aberrant or missing beat detection, which may have led to an advantage in AC. Regression analysis showed less point scatter and Bland Altman plots showed less bias with narrower limits of agreement with AC. However, outliers were still present with AC, making a potential erroneous result possible. The question arises as to why conventional artefact correction modalities do not appear to accurately reproduce this non-linear index especially with increasing artefact levels. It may not be possible to correctly predict lost beat placement based on formulas that do not consider the fractal, self-similar characteristics that DFA a1 is based on. Supporting this concept is that with increasing levels of artefact correction, DFA a1 showed higher values at uncorrelated level than at fractal and well correlated level. Past proposals for replacing missed beats have explored non-linear methods [21,39] and may be reconsidered. The underlying mechanism for the disparity between Polar H7 vs. ECG measurements is also unclear but may revolve around methodology of the preprocessing algorithms used to filter background noise and muscular contractile activity [40,41]. Since the DFA a1 index reflects interbeat fractal correlation properties, subtle changes in measuring the R wave peak could be responsible for the changes seen between recording devices [42]. 

## 5. Limitations and Future Directions

Although we chose the Kubios “Threshold” artefact correction option in the present study as “Medium”, other RR timing prediction limits could have been utilized (Very Low, Low, Strong, Very Strong). Recent data indicates that setting the “Threshold” to “Very Strong” may cause an excessive degree of interpolation [31]. That report recommended choosing any filter setting except “Very Strong” for adult data series analysis. Therefore, the “Medium” setting may represent an optimal balance between sensing a true artefact as opposed to mistakenly interpolating normal sinus variations. Sample size was relatively small for both the artefact related and device comparisons. Due to the need for the reference based HRV data to be free of artefact including atrial premature complexes (APCs), future studies may need substantial numbers of participants to obtain arrythmia free samples. Regarding direct device comparison, resting measurements were not done to determine bias between the Polar H7 and ECG during that condition. It is certainly possible that these bias results do not apply to resting data with high correlation, fractal patterns. Although recording device sample rates have been proposed as a possible factor in non-linear HRV bias [43], both the ECG and Polar H7 possess identical rates of 1000 Hz. If further study does confirm device related bias in the DFA a1 at high intensity ranges, adjustments in HRVT calculations may be necessary. In addition, modification of DFA a1 targets for constant power exercise sessions may be needed. An exciting development regarding chest belt recording revolves around that of artefact and arrhythmia identification. Upcoming generations of chest belt devices will possess the capability to display the ECG waveform, opening the possibility of proper correction of missed beats, noise and arrhythmia identification [44].

## 6. Conclusions and Practical Implications

Three major findings were observed in this study. First, bias induced by missed beat artefact correction depended on the correlation properties of the measured RR time series. The highest level of bias occurred at uncorrelated and anticorrelated values of DFA a1 resulting in an upward shift in the index. At fractal to correlated levels (DFA a1 near or above 1), minimal effects were seen. This is fortuitous since this range is present during resting and minimal exercise intensity, situations commonly studied. However, as exercise intensity rises and DFA a1 declines, artefact correction bias may substantially increase, potentially causing a falsely high DFA a1 reading at uncorrelated levels. If one were using this index to adjust training efforts based on power levels to avoid an uncorrelated DFA a1, substantial artefact could lead to misleading intensity targets. Nevertheless, despite the variable bias on the raw DFA a1 value, the HRVT determination did not appear to suffer greatly at low to moderate artefact levels (1% to 3%). This should be reassuring to investigators, coaches and athletes interested in using the index behavior either for aerobic threshold determination by means of an incremental ramp or internal load assessment during constant power exercise intervals. The finding of a chest belt recording device having a negative bias was unexpected. Studies using similar devices have shown reasonable concordance with high quality ECG derived HRV parameters but have mainly been compared at rest and/or contained some artefact in the RR series. In addition, there is the possibility that the downward Polar H7 bias combined with the upward artefact correction bias leads to values near the reference range. Given the suggestive but limited data on device related bias during exercise, further comparative study is recommended. In summary, levels of artefact correction commonly seen during exercise may cause some bias in DFA a1 measurement but should not lead to invalidating its usage with exercise threshold concepts.

## Figures and Tables

**Table 1:** Two-minute measurement windows derived from ECG recordings for DFA a1 no artefact (NA) vs. artefact containing (1, 3, 6%) data and correction method (automatic, AC, medium threshold, MC): Mean (±standard deviation, SD), Median, Minimum, Maximum, adjusted median difference (AMD) from DFA a1 vs. NA according to Hodges Lehmann method (*p*-value estimated by Wilcoxon paired testing), coefficient of determination (R^2^), Pearson’s r and Standard Estimate of Error (SEE) from paired data of DFA a1 NA vs. DFA a1 for each artefact condition and correction method.

	DFA a1 NA	DFA a1 1% AC	DFA a1 1% MC	DFA a1 3% AC	DFA a1 3% MC	DFA a1 6% AC	DFA a1 6% MC
Mean (±SD)	0.9518 (±0.404)	0.9512 (±0.4027)	0.9505 (±0.39)	0.9579 (±0.3993)	0.9569 (±0.3926)	0.9712 (±0.3953)	0.9677 (±0.3675)
Median	1.0251	1.0267	1.03385	1.0315	1.01605	1.014	1.02955
Maximum	1.5995	1.5986	1.6567	1.6039	1.6566	1.6041	1.633
Minimum	0.2171	0.2212	0.2402	0.2281	0.291	0.2208	0.3202
AMD (vs. NA)		0.0012 (*p* = 0.002)	0 (*p* = 0.999)	0.0048 (*p* = 0.0001)	0.0111 (*p* = 0.15)	0.0146 (*p* = 0.0001)	0.0223 (*p* = 0.01)
R^2^ (vs. NA)		0.999	0.977	0.997	0.960	0.983	0.962
Pearson’s r (vs. NA)		0.999	0.989	0.998	0.980	0.991	0.981
SEE		0.013	0.059	0.023	0.079	0.052	0.072

**Table 2:** Comparison of ECG derived heart rate at HRVT for 10 participants with no baseline artefact (NA), across each artefact condition and correction method. Mean (±standard deviation, SD) in last row. * denotes *p* ≤ 0.05 on t testing HRVT NA vs. HRVT for each artefact condition and correction method.

	HRVT NA	HRVT 1% AC	HRVT 1% MC	HRVT 3% AC	HRVT 3% MC	HRVT 6% AC	HRVT 6% MC
	155.2	155.3	156.1	156.6	155.9	159.8	156.3
	185.2	185.4	185.7	185.9	184.6	186.0	184.7
	125.9	126.3	125.7	125.9	125.3	126.5	124.3
	137.5	137.9	137.9	137.7	137.6	138.3	137.9
	137.4	137.5	138.1	137.5	138.3	137.5	138.7
	162.6	163.0	163.0	163.2	164.2	163.3	165.0
	175.4	175.0	176.3	175.8	176.7	175.7	179.0
	133.8	134.7	133.3	135.6	134.3	138.2	137.6
	170.6	170.2	170.5	170.4	169.7	171.1	171.2
	174.6	174.0	174.7	173.9	175.0	174.3	175.0
Mean (±SD)	155.8 bpm (±20.9)	155.9 bpm (±20.6)	156.1 bpm (±21.0)	156.3 bpm (±20.7)	156.2 bpm (±20.9)	157.1 bpm * (±20.4)	157.0 bpm (±21.0)

**Table 3:** Comparison of heart rate at HRVT between ECG vs. Polar H7 data both using automatic artefact correction (AC). Percent artefact for all data calculated over the HRVT measurement range below 5%. Mean (±standard deviation, SD) in last row. * denotes *p* ≤ 0.05 on paired t testing.

	HRVT ECG AC	HRVT Polar H7 AC
	155.2	152.3
	185.2	182.9
	125.9	124.6
	137.5	135.1
	160.0	156.4
	137.4	137.5
	162.6	154.6
	160.3	157.1
	171.0	166.0
	175.4	164.0
	133.8	129.0
Mean (±SD)	154.9 bpm (±19.0)	150.9 bpm * (±17.6)

## Data Availability

The raw data supporting the conclusions of this article will be made available by the authors, without undue reservation.
